# Inter-Ethnic Variations in the Clinical, Pathological, and Molecular Characteristics of Wilms Tumor

**DOI:** 10.3390/cancers16173051

**Published:** 2024-09-01

**Authors:** Kia Teng Lim, Amos H. P. Loh

**Affiliations:** 1 Ministry of Health Holdings, Singapore 139691, Singapore; kiatenglim@u.nus.edu; 2 VIVA-KKH Paediatric Brain and Solid Tumour Programme, Children’s Blood and Cancer Centre, KK Women’s and Children’s Hospital, Singapore 229899, Singapore; 3 SingHealth-Duke NUS Global Health Institute, Duke-NUS Medical School, Singapore 169857, Singapore; 4 Department of Paediatric Surgery, KK Women’s and Children’s Hospital, Singapore 229899, Singapore

**Keywords:** Wilms tumor, inter-ethnic variations, loss of heterozygosity, anaplasia, favorable histology

## Abstract

**Simple Summary:**

Wilms tumor is the most common pediatric primary renal malignancy globally but exhibits significant variations in epidemiological, clinical, and molecular aspects among different populations. Wilms tumor has been shown to occur at lower incidences, younger ages, and earlier stages of disease with higher incidences of favorable histology among Asians compared to Caucasians. Despite a worldwide convergence between the two main management approaches, it is not known if these are generalizable to all ethnic populations. This paper summarizes the current literature on the inter-ethnic variations in the clinical, pathological, and molecular characteristics of Wilms tumor.

**Abstract:**

Wilms tumor is the commonest primary renal malignancy in children and demonstrates substantial inter-ethnic variation in clinical, pathological, and molecular characteristics. Wilms tumor occurs at a lower incidence and at a younger age in Asians compared to Caucasians and Africans. Asians also present at an earlier stage of disease, with a higher incidence of favorable histology tumors and a lower incidence of perilobar nephrogenic rests compared to Caucasians, while African children present with more advanced disease. Studies have implicated population differences in the incidence of WT1 mutations, loss of imprinting of the IGF2 locus, and loss of heterozygosity of 1p/16q, or 1q gain as possible bases for epidemiological differences in the disease profile of Wilms tumors in various ethnic groups. Yet, evidence to support these associations is confounded by differences in treatment protocols and inequalities in the availability of treatment resources and remains limited by the quality of population-based data, especially in resource-limited settings.

## 1. Introduction

Pediatric renal tumors account for 3–11% of childhood cancers worldwide [1]. Wilms tumor, also known as nephroblastoma, is the most common pediatric primary renal malignancy, despite only comprising 5% of childhood malignancies [2], affecting 1 in 10,000 children. As with other pediatric embryonal tumors, they arise from abnormalities of embryonic development with disrupted nephrogenic differentiation thought to be the key pathogenic process giving rise to rapid cellular proliferation [3].

First described by Thomas F. Rance in 1814, with further characterization of its histology by German surgeon and pathologist Carl Max Wilhelm Wilms in 1899, over a century of clinical research since then has led to the refinement of multimodal treatments for Wilms tumor that have achieved excellent survival outcomes in the majority of cases [4]. Two large collaborative groups have been the main drivers behind the evolution of Wilms tumor management—the National Wilms Tumor Study Group (NWTS) in the United States and the International Society of Pediatric Oncology (SIOP) in Europe. The cumulative efforts of both groups have transformed Wilms tumor from a largely fatal disease with 70% survival [5] to one with a current overall 5-year survival rate of 92% in developed countries, although survival outcomes lag behind in other less developed parts of the world with fewer resources [6]. Equally apparent is the omission of large populations outside Europe and North America from collaborative trials with corresponding gaps in knowledge and awareness in these resource-limited settings [7].

**Figure 1:**
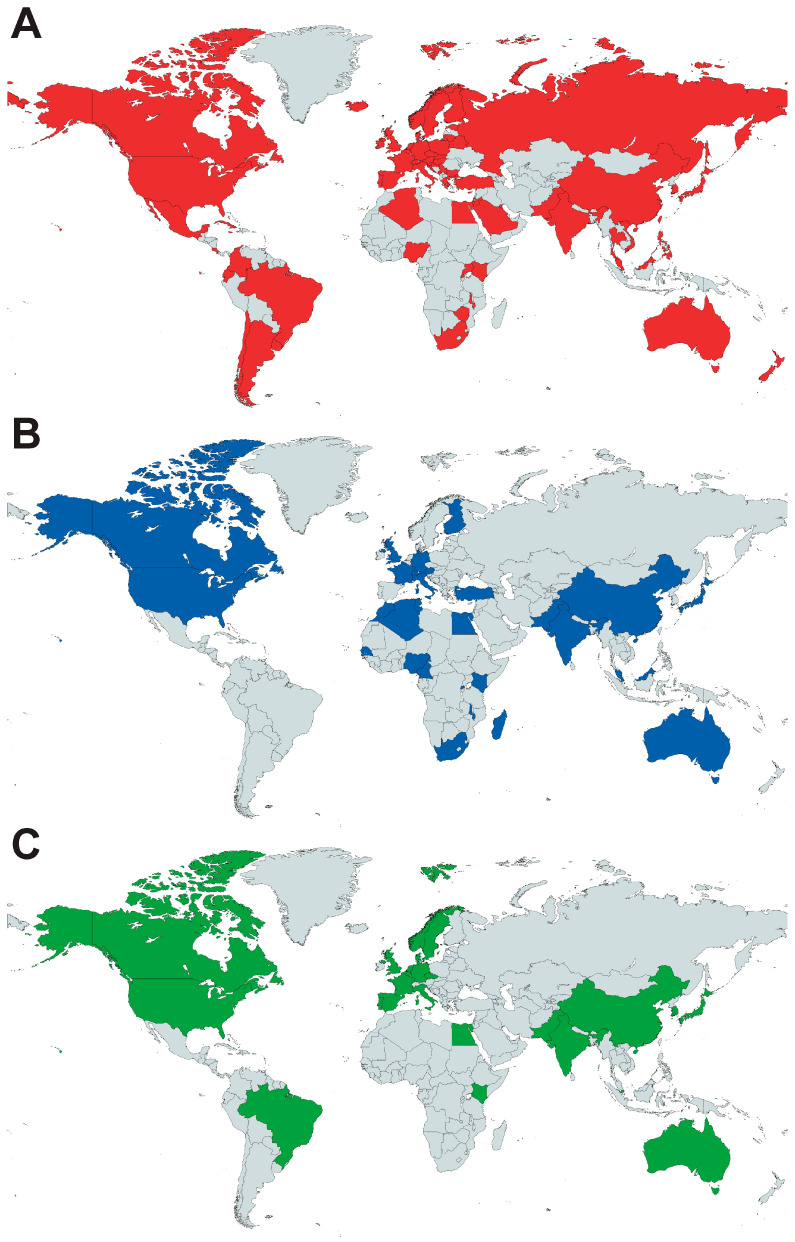
Geographical distribution of countries with published population-specific data describing (**A**) demographic, (**B**) clinicopathological, or (**C**) molecular characteristics of Wilms tumor (created with mapchart.net). Countries included are in (**A**) Africa (Algeria, Egypt, Kenya, Malawi, Mauritius, Nigeria, South Africa, Uganda, Zimbabwe), Asia (Bahrain, China, Cyprus, India, Israel, Japan, Jordan, Kuwait, Malaysia, Pakistan, Philippines, Saudi Arabia, Singapore, South Korea, Taiwan, Thailand, Turkey, Vietnam), Europe (Austria, Belarus, Belgium, Bulgaria, Croatia, Czech Republic, Denmark, Estonia, Finland, France, Germany, Greece, Hungary, Iceland, Ireland, Italy, Lithuania, Malta, Montenegro, Norway, Netherlands, Poland, Portugal, Russia, Serbia, Slovakia, Slovenia, Spain, Sweden, Switzerland, United Kingdom), North America (Canada, Costa Rica, Cuba, Jamaica, Mexico, United States of America), Oceania (Australia, New Zealand), South America (Argentina, Brazil, Chile, Colombia, Ecuador, Uruguay); (**B**) Africa (Algeria, Cameroon, Egypt, Kenya, Madagascar, Malawi, Morocco, Nigeria, Rwanda, Senegal, South Africa, Tunisia), Asia (China, India, Japan, Malaysia, Pakistan, Singapore, Turkey), Europe (Austria, Finland, France, Germany, Italy, Switzerland, United Kingdom), North America (Canada, United States of America), Oceania (Australia); (**C**) Africa (Egypt, Kenya), Asia (China, India, Japan, Korea, Pakistan, Singapore), Europe (Sweden, United Kingdom), North America (Canada, United States of America), Oceania (Australia), South America (Brazil).

Most recently, treatment regimens for Wilms tumor have introduced molecular biomarkers for clinical risk stratification, in particular, loss of heterozygosity (LOH) of chromosomal loci 1p/16q and 1q [8]. However, despite being treated with similar treatment protocols, Asian patients have been shown to have better survival outcomes compared to non-Asians, independent of LOH status and the presence of nodal metastasis [9]. Asian patients have also been observed to have more favorable clinical characteristics—fewer unfavorable histology tumors and lower-stage disease—which likely account for overall better survival [10]. With current cooperative group protocols being developed largely from findings in Caucasian populations, there is limited consideration given to the effect of ethnic variations in clinical and histopathological characteristics on the treatment of Wilms tumor [11]. This article reviews current evidence on the inter-ethnic variations in the epidemiology, histopathology, genomics, and evolution of the treatment strategies for Wilms tumor (Figure 1), identifying crucial gaps in our current knowledge and translational application. In this paper, the geographical definition of Asian populations was in concordance with the M49 standard published by the United Nations Secretariat [12].

## 2. Epidemiology of Wilms Tumor

### 2.1. Incidence

Reports from the 1990s first described inter-ethnic variations in the incidence of Wilms tumors [13,14]—Asian Wilms tumors had lower incidences and tended to occur at a younger age, with up to 40% occurring in infants less than 1 year old, compared to Caucasian and Black populations with peak incidences around the second year of life. In a study of Wilms tumors among 7658 children in Britain, the incidence of Wilms tumors among Asian children was half that among Caucasians: 4% among 366 Asian children versus 8% among 6783 Caucasian children [15]. Another study by Axt et al. analyzing 2515 patients less than 20 years of age from the Tennessee Cancer Registry between 1999 and 2008 revealed that Wilms tumor was 79% more likely to occur among African-American patients than White Americans [16]. More current data from the Surveillance, Epidemiology, and End Results (SEER) Program suggests that socioeconomic status (SES) may be a modifier of disease incidence—the SES quintile has been shown to be positively associated with incidence among Hispanics but is negatively associated among Asian/Pacific Islanders [17].

The reduced incidence of Wilms tumor in the Asian population has been hypothesized to be due to the loss of imprinting (LOI) for the Insulin-like Growth Factor 2 gene (IGF2) occurring at a lower frequency in Asians [18]. Spreafico et al. highlighted that in the United States of America (USA), African-American children have the highest incidence (9.7 per million) compared to Asians who have the lowest incidence (3.7 per million), although the inter-ethnic variation in worldwide incidences is interpreted with some caution due to the shortage of population-based cancer registries in low- and middle-income countries [19].

### 2.2. Gender Distribution

Another area where epidemiological characteristics differ significantly by ethnicity is gender distribution. Wilms tumor has been known to be more common among girls. A recent review by Nakata et al., including more than 300 childhood cancer registries worldwide, showed that globally, Wilms tumors had a male/female sex ratio of 0.7 (95% C.I.: 0.8–0.9) [20]. However, the included registries significantly over-represented Western populations, with only a 1.7–4.6% coverage of South, Southeast, and East Asian populations in comparison to coverage rates of 99.4% in North American populations [21]. Yet, this is unsurprising due to the lack of robust population-based registries in low- and middle-income countries, which predominate in Asia and Africa [22]. In contrast, multiple series from Asian populations have indicated an equal gender distribution, and in some, a male predominance [9,23,24]. Taken together, these studies could suggest that differences in gender predominance for Wilms tumors between different ethnicities could have been masked by the lack of their representation in registry-based data and earlier historical studies.

It has also been suggested that patient gender may influence disease outcome, and again that ethnicity may be a modifier affecting this potential association. In a comparative study between children with Wilms tumors in the UK and Japan over a 20-year period, the female sex was shown to be significantly associated with an increased risk of death in the UK but not in Japan [25]. Yet, a review by Groenendijk et al. concluded that across the UK Children’s Cancer and Leukemia Group (UKCCLG), the North American Children’s Oncology Group (COG), and SIOP studies, there was no association between gender and tumor recurrence [26]. Nevertheless, there is a paucity of evidence on the inter-ethnic variation in gender differences for Wilms tumor, and again, with the underrepresentation of non-Caucasian races in these studies, the true relationship between gender and outcome in these ethnic groups may not yet be fully understood.

### 2.3. Age of Clinical Presentation

The earliest studies cite the median age of onset for Wilms tumor as 3 years, with girls being diagnosed 6 months later than boys [14]. Consistent with this, a more recent study by Doganis et al. comparing Wilms tumor trends across Europe and the USA found that females presented later—with a female predominance in children aged 5 to 14 years versus that of males in infancy [27]. Similar observations have been reported in other ethnic groups in North Africa and the Middle East. A large-scale retrospective cohort study based in Egypt reported a mean age of presentation of 5.25 years, with a similar female predominance [28]. Understanding the differences in the average age of presentation in different ethnic groups is important due to the association of age with survival outcomes. Several long-term cohort studies have concluded that age <2 years was a favorable prognostic factor for event-free survival (EFS) and overall survival (OS) [29,30], while age >4 years was recognized as an adverse prognostic factor [31,32,33,34].

Multiple studies have consistently indicated an earlier peak age of diagnosis of Wilms tumor in Asian children, typically in the second year of life [35]. This pattern and its association with outcomes has been best described in Japanese populations. Nakata et al. compared over 1500 patients with Wilms tumors in the United Kingdom (UK) and Japan from 1996 to 2015 [25], where Japanese patients were found to have a significantly younger median age at diagnosis than patients in the UK (28 months versus 39 months), with no differences in five-year OS or EFS between both populations. Among Japanese children, the peak age at diagnosis was 12 to 18 months (1 to 3 years), compared with a broader, bimodal peak among UK children ranging between 12 and 42 months (i.e., 1 to 3.5 years). Similar findings were noted in another study by Fukuzawa et al. [36], in which age at diagnosis among Caucasian patients followed a bimodal distribution, while Asian patients showed an unimodal age distribution. However, the study was limited by the significantly smaller numbers of Asian patients recruited in the study (148 Asians versus 5002 Caucasians). Age distribution among other Asian populations outside Japan has been less well studied. A 22-year study of Malaysian children found that the majority of the patients were below 2 years of age [37]. Beyond these few series, the clinical significance of the age of presentation across different races remains largely unexplored. Nevertheless, the currently available evidence does indicate strongly that Wilms tumor occurs at a younger age in East Asian populations, which is of particular interest given the known association of younger age with favorable outcomes, which is indeed also observed in these patients. Notably, the age distributions of non-Wilms tumors in Asian populations are even less well studied. This is important given that the age-based treatment algorithm of the SIOP Renal Tumor Study Group assumes an increased incidence of non-Wilms tumors under 6 months of age, advising upfront nephrectomy for children below this age. However, this cut-off has not been verified in non-Western populations, though SIOP protocols are used in many Asian countries. More studies are needed to evaluate the percentage of children in Asian countries aged less than 6 months that receive upfront nephrectomies for Wilms tumor while being treated on a SIOP protocol.

## 3. Clinicopathological Characteristics

### 3.1. Stage of Wilms Tumor at Presentation

Disease stage and tumor histology critically determine the choice of systemic treatment in Wilms tumor. Favorable histology (FH) tumors with lower stages 1 and 2 are treated with vincristine and dactinomycin (VD) or combinations of actinomycin and doxorubicin (AD), whereas unfavorable histology (UH) tumors with stages 2 to 4 are treated with cyclophosphamide, ifosfamide, carboplatin, and etoposide, in addition to radiotherapy; stage 5 UH tumors are treated with vincristine, dactinomycin, and doxorubicin for 6 to 12 weeks [38]. Despite advances in treatment, advanced stages of Wilms tumor remain associated with higher risks of relapse and increased morbidity [39].

Various studies have attempted to characterize the inter-ethnic variation in the stage of disease at presentation—most series suggest that African children tend to present with distant disease more frequently than White children [16], while Asian patients have lower-stage disease at presentation than both these other populations, which may contribute to their better outcomes [10]. However, most attempts to compare disease stage at presentation across ethnic groups are confounded by differences in practice context and health standards [40]. In a comparative study on race disparities from a North American statewide cancer registry, more Black children with Wilms tumor presented with distant disease than White children (25% versus 15%), with Black males showing the lowest survival compared to other gender and ethnic subgroups [16]. In Nigeria, children were shown to present with Wilms tumors at late stages, the majority in stages 3 to 4 [41]. Similarly, in Malawi, patients were found to present at advanced stages [42]. In an analysis of Wilms tumors across 20 years in a multiracial population in Southeast Asia, Asian children presented at significantly earlier stages (majority at stage I to II) compared to non-Asians (majority at stage III and beyond), with lower rates of distant metastases despite having similar rates of LOH of 1p and 16q [9]. Notably, other Asian series have also reported the same trend. In a single-center series from Rawalpindi, Pakistan, most cases presented early in stages I to II [43]. Similarly, a systematic review of 1170 patients in India showed that the majority of patients there presented early in stages I to II [44].

One of the postulations for the discrepancy in disease stage at presentation includes differences in access to healthcare services, leading to more delayed presentations in low- and middle-income countries (LMICs) compared to high-income countries (HICs) [45]. In a retrospective study of children with Wilms tumor in Kenya, it was found that most of them presented at advanced stages—over 80% with stage III to IV disease [46]. Another 15-year retrospective study by Nasir et al. showed that the majority of Nigerian patients (74.3%) presented with advanced disease at stage III to IV [47]. Similarly, Alakaloko et al. conducted a retrospective study in Nigeria, which also revealed that the majority of their patients presented with stage III disease [48]. In a retrospective study of a South African cohort by Stones et al., most patients presented at stage IV [49]. Interestingly, in a comparison of presentations and outcomes of childhood cancers between an LMIC center versus an HIC center, it was found that the time from onset of first symptoms to initial presentation at the hospital was significantly longer in the South Egypt Cancer Institute (SECI) in Egypt compared to University Hospital of Cologne-Uniklinik Koln in Germany, but that this was not confounded by geographical distances, which was similar in the two settings [50]. Yet, patients in the SECI needed longer travel time to reach the treatment center, showed less compliance to therapy, and correspondingly relapsed earlier; there were more deaths from infectious conditions rather than disease-related deaths.

In summary, although fair comparisons of differences in the stage of disease at presentation remain challenging, the prevailing trend in the available data suggests that children of Asian ethnicity tend to present in earlier stages of their disease, while children of African ethnicity present with more advanced disease. With some studies indicating that the association between the stage of disease at presentation and ethnicity may be independent of the healthcare setting, this may suggest the presence of underlying biological factors related to disease behavior, which may account for this inter-ethnic variation in disease presentation [16].

### 3.2. Histological Anaplasia

The presence of anaplasia in Wilms tumor is termed unfavorable histology (UH) and is a known prognostic factor for relapse [51]. UH is defined as the presence of cells with large hyperchromatic nuclei and multipolar mitotic figures and is associated with resistance to chemotherapy, leading to poorer outcomes [52]. Conversely, favorable histology (FH) Wilms tumors do not display anaplasia and are associated with lower-stage disease and excellent long-term prognosis [53], with a 4-year survival rate ranging from 95 to 100% for stage I to III tumors [54]. In the cohort study of 48 patients in Singapore, the proportion of patients with UH was significantly higher among non-Asian than Asian patients, despite both subgroups having a similar LOH status of 1p and 16q [9]. In a recent Japanese study, patients included in the Japan Wilms Tumor Study Group (JWiTS) were found to have a significantly lower incidence of anaplasia compared to patients in the USA recruited in the NWTS-5 trial [55], with only 4.9% of Japanese patients having UH compared to 10.8% of NWTS-5 patients [56]. The largest retrospective study of Wilms tumors in India reported that tumor histology was the most significant factor in the prediction of survival, and a higher rate of FH (80.3%) was also noted among Indian patients compared to those from NWTS studies [57].

Anaplasia has been commonly associated with TP53 loss [58], while mutations in WT1 and epigenetic changes—particularly loss of imprinting (LOI)—at the Insulin-like Growth Factor II (IGF2)/H19 locus have been associated with FH [59]. It is hypothesized that the presence of TP53 mutations may contribute to the resistance of Wilms tumors to chemotherapy, which accounts for the poorer prognosis of UH [60]. In a JWiTS study of bilateral tumors in Japan, a significantly higher incidence of WT1 abnormalities was found among Japanese patients in comparison to Caucasian patients [61]. While this could potentially account for greater rates of FH among Asians, other evidence supporting associations between race and histology are inconsistent and largely unexplored. The same group of investigators studying sporadic Wilms tumors then found the frequencies of WT1 abnormalities to be similar between their Japanese cohort and Caucasian populations [62]. Similarly, in another study based in the USA, among children with UH or relapse Wilms tumors, there were no significant differences found in the distribution of UH and FH tumors between Black and White patients [63].

### 3.3. Histological Subtype

Classic Wilms tumors are triphasic, consisting of blastemal, stromal, and epithelial cell types [64]. The proportion of the three cell types remaining after chemotherapy bears prognostic relevance—blastemal components are the least differentiated and harbor the most malignant and aggressive behavior [65]. Contrastingly, epithelial-predominant FH Wilms tumors have been associated with excellent outcomes, less invasiveness, and metastatic potential [53]. Tumor histological subtype thus contributes towards risk stratification of Wilms tumors—the current SIOP classification defines blastemal-dominant histological subtype tumors as high risk [66], as blastemal predominance is associated with increased risk of mortality, along with diffuse anaplasia, low differentiated epithelial, and regressive subtypes [67]. Data from the SIOP93-01/GPOH study affirmed the significantly lower association with blastemal-predominant tumors (82% EFS) when compared to other histological subtypes (93–100% EFS) [68].

In the early 1990s, Cheah et al. were amongst the first few to observe inter-ethnic variation in histological features among Asian patients with Wilms tumors and reported a higher proportion of epithelial-predominant tumors and a lower percentage of blastemal-dominant tumors among Malaysian children in their 22-year follow-up study [37]. In contrast, another study from sub-Saharan Africa reported that blastemal dominance was the most common subtype [69]. However, with such limited data available, no clear and significant associations between ethnicity and histology subtype have been established.

### 3.4. Nephrogenic Rests

The poor prognosis associated with blastemal predominance histology has been hypothesized to be related in part to the presence of nephrogenic rests (NRs) [70]. NRs are regarded as pre-malignant lesions derived from embryogenic growth-arrested metanephric blastemal cells [71]. First described by Beckwith et al. in 1990 as a new classification system, two major categories of NRs were recognized—perilobar nephrogenic rests (PLNRs), which are sited in peripheral locations and display blastemal-predominant histology in early lesions, and intralobular nephrogenic rests (ILNRs), which are randomly distributed in the kidney and usually have stromal-predominant histology [72]. ILNRs are also noted to arise earlier than PLNRs [73]—specifically, ILNRs are noted in patients at a median age of 23 months, whilst PLNRs are usually discovered only at a median age of 36 months of age [74]. Consistent with the pathogenesis of NRs, Breslow et al. conducted a review on more than 7000 patients analyzed across the NWTS-3,4,5 studies, revealing that ILNR was associated with a mean age of Wilms tumor diagnosis that was earlier by 18 months [75].

Beckwith was also among the first to identify variations in the prevalence of NR across different races—PLNR seemed to be rarely encountered in Asian children compared to the Western population, which was thought to account for the lower incidence and lower age of onset for Wilms tumors among Asians [76]. This is also consistent with the current literature, where Caucasian Wilms tumors were found to have higher rates of PLNR (20%) compared to Asian-American children (7%) and Japanese children (2%) [74]. In another study on precursor lesions of Wilms tumors among Indian children, Mishra et al. observed that PLNR was completely absent in all 127 cases in their series, with ILNR being associated with 45.3% of the WT samples [77].

Even though the significance of inter-ethnic variation in NRs remains unclear, the implications of NRs on clinical management are still evolving. Currently, no treatment guidelines stratify patients by NR status for therapeutic escalation or reduction. However, a recent systematic review by Brown et al. showed that NRs detected in specimens after radical nephrectomy are associated with a 3% increased risk of developing metachronous tumors, suggesting that there may be a role for prolonged surveillance of the contralateral kidney in patients with sporadic Wilms tumors associated with NRs [78].

The presence of NRs is also known to be associated with bilateral Wilms tumors, which have a poorer prognosis—approximately 28 to 40% of unilateral Wilms tumors are associated with NR, compared to 90 to 100% of bilateral Wilms tumors [79]. In unilateral Wilms tumors, NRs are usually only found in histopathology specimens post-resection, whereas, for bilateral Wilms tumors, NRs could be large enough to be detected on imaging [80]. The low frequency of NRs in Asian patients with sporadic Wilms tumors appears to be congruent with the similarly low frequency of bilateral Wilms tumors. This observed co-occurrence aligns with known clinical associations of bilateral Wilms tumors and NRs [75]. An ontogenic model of Wilms tumors based on gene expression patterns also proposes that bilateral Wilms tumors may belong to the same molecular subgroup as Wilms tumors that arise from NRs, with both associated with developmental events occurring in the early metanephric mesenchyme [81].

### 3.5. Bilateral Wilms Tumor

In accordance with the NWTS studies, metachronous bilateral Wilms tumors are shown to have lower long-term survival rates, ranging from 49% to 80% [82], compared to unilateral Wilms tumors, with disease-free survival rates ranging from 70% to 90% and higher rates of renal insufficiency [83]. The incidence of end-stage renal failure (ESRF) in a France-based study by Sudour et al. was shown to be 0.6% amongst those with unilateral Wilms tumors compared to 11.5% in bilateral Wilms tumors [84].

Across numerous studies, bilateral Wilms tumors were found to have a female predominance and an earlier age of presentation compared to unilateral tumors [85]. The overall incidence of bilateral Wilms tumors is reported as 5 to 8% in most cohorts [80,86,87,88]; however, there is mixed evidence on their incidence in populations from Asia and Africa [87,89,90,91,92]. In a cohort from Singapore, no Asian patients in the 10-year series had bilateral tumors [9]. Contrastingly, the Japan Wilms Tumor Study (JWiTS) group demonstrated in their 15-year study that the incidence of bilateral Wilms tumors was similar to that of the general population at 3 to 8% [93]. In the narrative review by Apple et al., Black children were more predisposed to developing bilateral Wilms tumors, possibly attributed to their increased susceptibility to germline mutations [94]. Other than the publications of population-based registry data from Japan, most other papers from Asia and Africa, being single-center series, might not have adequately reflected the true population incidences of bilateral Wilms tumors.

Bilateral Wilms tumors have been shown to harbor genetic associations that vary between different ethnic groups. A high incidence of bilateral disease is observed in patients who demonstrate loss of imprinting (LOI) at the 11p15 chromosome, affecting the IGF and H19 genes [80]. Fukuzawa et al. reported that the incidence of IGF2 LOI in Japanese children was significantly lower than that in Caucasian children [36], corresponding with the lower incidence of bilateral Wilms tumors in the Japanese cohort. Conversely, Japanese children with bilateral Wilms tumors instead were found to have a higher rate of WT1 mutations—among the 355 children analyzed in the JWiTS group, the WT1 mutation was found in 78% of those with bilateral Wilms tumors [93], which is significantly higher than observed in their Caucasian counterparts with bilateral Wilms tumors (17 to 38%) [61]. Taken together, these observations point towards potential differences in the epigenetic basis for bilateral disease in different ethnic groups.

### 3.6. Genetic Mutations

Various somatic mutations have been known to be associated with Wilms tumor—in particular, somatic WT1 mutations are observed in 10 to 20% of Wilms tumors in general [26]. Oue et al. have suggested that it could be pertinent to conduct WT1 mutation analysis with bilateral Wilms tumors, as mutations in WT1 have also been implicated in renal pathologies that may eventually lead to ESRF [93,95]. Currently, functional studies demonstrate how WT1 gene mutations induce dysfunction in renal podocytes or progenitors [95]. Additionally, studies using mice models have hypothesized that reduced WT1 expression may lead to mesangial sclerosis of the glomeruli and eventual ESRF, implicating that WT1 deletion may contribute to the development of ESRF [96,97]. This observation is consistent with findings from Grigoriev et al.’s clinical study, where across more than 9000 subjects enrolled in the NWTS 1 to 5 trials, nearly half of the chronic kidney disease (CKD) patients had a WT1-associated mutation or congenital anomaly [98]. Correspondingly, case reports of Asian patients with WT1 mutations, renal dysfunction, and unilateral Wilms tumors demonstrate a similar clinical picture [99,100], but this association has not been described in Asian patients with bilateral Wilms tumors, presumably because of the low incidence in Asian populations.

Despite WT1 being well studied as a tumor suppressor gene, its role in non-WT1-mutated Wilms tumors and the corresponding implications on prognostic outcome remain ambiguous [101]. Of note, some studies suggest that certain WT1 anomalies could in fact be linked to more optimal outcomes in some ethnic groups. In a Japanese study on unilateral Wilms tumors, the majority of Wilms tumors with WT1 anomalies showed stromal predominance, while the majority of Wilms tumors without WT1 mutations showed blastemal-predominant histology [102]. It was hypothesized that Japanese Wilms tumors may be characterized by a higher incidence of WT1 deletion, which could contribute to the lower incidence of Wilms tumors in Japanese children as compared to Caucasian populations [103]. However, it remains challenging to compare the incidence rates of WT1 mutations in Wilms tumors across different races due to WT1-mutated sporadic or unilateral Wilms tumors having a considerably low incidence of less than 10% [102].

The LOI of the IGF2 gene is known to be the most common epigenetic alteration in Wilms tumors and has been noted in approximately half of all Wilms tumors [104]. In a large-scale study of over 110 samples in over 780 Japanese patients, the incidence of IGF2 LOI was found to be lower in Japanese children compared to Caucasian children, supporting the hypothesis that lower IGF2 incidence contributes to lower WT incidence among the Japanese [62]. Similarly, in an inter-ethnic comparative study on pediatric patients with Beckwith–Wiedemann syndrome (BWS)—which is known to predispose patients to Wilms tumors—the frequency of isolated H19-DMR hypermethylation was found to be significantly lower in Japanese patients with BWS than North American and European patients [105].

Apart from the IGF2 gene, mutations in the WTX and CTNNB1 genes are also known to be implicated in the pathogenesis of Wilms tumors. However, inter-ethnic variations and associations with anomalies of these two genes have not been studied as thoroughly [106,107]. The study by Haruta et al. found that the incidences of WTX mutations and CTNNB1 mutations in Wilms tumor patients were much lower among Japanese patients compared to Western patients. They noted a 25.7% incidence of WTX mutations in Japanese patients versus 9.4% to 22.2% in Caucasians and 20.8% of CTNNB1 mutations in Japanese patients versus 16.7% to 23.5% in Caucasians [62].

Several studies have identified other genetic alterations that are correlated with advanced stages of disease, such as copy number variations in TOP2ATopoisomerase IIα [108] and the expression of CRABP2 [109] and IGFR-1 [110]. Recently, Mahamdallie et al. also identified four new Wilms tumor predisposition genes after analyzing lymphocyte DNA from 890 patients with Wilms tumors—TRIM28, FBXW7, NYNRIN, and KDM3B mutations [111]. However, little is known about the inter-ethnic variations of these genetic mutations.

### 3.7. Copy Number Variations

In recent years, treatment protocols for Wilms tumors have incorporated risk stratification systems incorporating the LOH of chromosomes 1p and 16q, and 1q gain [112]. Co-LOH at chromosomes 1p and 16q was studied by the COG as a risk biomarker to intensify chemotherapy and radiotherapy [113,114] based on NWTS-5 data that identified the co-LOH of chromosomes 1p or 16q to be significantly associated with increased risk of relapse and death in FH Wilms tumors. The AREN0532 and AREN0533 studies, which included more than 2500 patients, showed that those with LOH at 1p/16q had greater 4-year EFS compared to those from the NWTS-5 study, even after excluding patients who may have been affected by changes in tumor staging definitions—87.3% in patients with LOH 1p/16q and stage I/II disease and 90.2% for patients with LOH 1p/16q and stage III/IV disease versus 68.8% in all patients with stage I/II disease and 61.3% in all patients with stage III/IV disease in the NWTS trial [8]. Furthermore, the utility of accounting for LOH 1p/16q becomes evident from the COG trial, where augmentation of chemotherapeutic regimens was shown to further improve outcomes across patients of all disease stages. Notably, and unsurprisingly, the racial composition of the study cohort was similar to NWTS-5 with no statistically significant difference, with Caucasians comprising the majority—67% White (versus 71% in NWTS-5) and 19% Black patients (versus 18% in NWTS-5); the race of the remaining 13% of patients was recorded as unknown (versus 12% recorded as other races in NWTS-5).

However, studies examining LOH at 1p and 16q have revealed varied consistency in their prognostic association across various ethnic groups. It has been hypothesized that variations in the prognostic values of LOH may be due to the inter-ethnic variations in study populations [115]. The evidence behind molecular risk stratification in Wilms tumor has been largely derived from studies on Caucasian populations [116], with the impact of LOH as prognostic factors and features of treatment stratification being relatively unexplored in other ethnic groups. In a meta-analysis of 10 studies involving 3385 patients, it was found that LOH at 16q was significantly associated with relapse; however, with the 10 studies mainly from Europe and North America, the authors suggested that the present collective evidence was insufficient to determine if factors like race affected the incidence of LOH 16q and hence, relapse risk [117].

Nevertheless, even without large-scale head-to-head comparisons, current evidence from the limited single-center studies of Asian patients with Wilms tumors does not show a consistent association of an LOH of 1p or 16q with clinical risk features and survival outcomes. In a Singapore-based cohort, microsatellite profiling showed no significant difference in the incidences of an LOH of 1p or 16q among Asians and non-Asians; among Asian patients (which comprised most of the study cohort), an LOH of 1p or 16q was not associated with any histological characteristics, stage of disease at presentation, or clinical outcomes [9]. In a retrospective analysis of 175 Wilms tumor samples from Beijing Children’s Hospital, the frequency of an LOH of 1p or 16q was significantly higher in children with advanced disease stages but was not correlated with lymph node metastasis; only an LOH of 1p was significantly associated with progression-free survival, while an LOH of 16q was not [118]. Conversely, in a Korean study of over 100 patients with FH Wilms tumors, an LOH of 16q but not 1p was significantly associated with poorer 3-year EFS [115]. Yet, another study of Indian patients found no significant differences in the distribution of 16q loss or 1q gain according to relapse status or stage of disease at presentation [119]. Few studies outside of North America have studied the prognostic association of a co-LOH of 1p and 16q in Wilms tumor patients. The above study from Singapore found that a co-LOH of 1p and 16q was not associated with survival outcomes [9]. A retrospective analysis of Egyptian patients also did not find that patients with a co-LOH of 1p and 16q had significantly decreased 3-year OS compared to those with isolated 1p or 16q loss [120]. These studies have questioned the validity of a co-LOH of 1p and 16q as a treatment risk stratifier in these populations. Variations in treatment approaches, standards of supportive care, and laboratory methods to determine LOH make it challenging to draw broad conclusions from the existing data and will require coordinated protocols across countries outside Europe and North America to study this more definitively.

Beyond an LOH at 1p and 16q and 1q gain, several studies in non-Western populations have also explored genetic aberrations of 11p in patients with Wilms tumors. In the 1980s, Dao et al. first showed the frequent presence of 11p alterations in most Wilms tumors [121]. An LOH of the 11p13 chromosomal locus corresponding to the WT1 gene [122], and an LOH of the 11p15 chromosomal locus corresponding to the IGF2 gene were subsequently described [123]. Anomalies involving the WT1 gene at 11p13 have been known to be associated with ILNR, while an LOI at 11p15 was known to be associated with PLNR [124]. Some inter-ethnic variations in the frequency of 11p aberrations have been observed, albeit of inconclusive clinical significance. In the 1990s, a comparative study showed that there were no significant differences in the distribution of 11p13 anomalies between Japanese versus American or British patients with Wilms tumors [125]. Subsequently, in the 2000s, Nakadate et al. showed in their study of 105 Japanese children that Japanese people had a higher incidence of LOH limited to 11p13 and a lower incidence of LOH at 11p15 than Caucasians [102]. Similarly, Sigamani et al. showed that an LOH at 11p15 occurred at a lower incidence among Indians compared to Western cohorts, while the incidence of an LOH at 11p13 in the Indian population was similar to the current literature [126], which is consistent with the known lower incidence of PLNR among Asians. It may be possible that population differences in molecular characteristics predisposing to particular clinical characteristics could account for epidemiological differences in disease phenotype; however, the quality of the current evidence limits our confidence to draw such conclusions.

## 4. Conclusions

**Figure 2:**
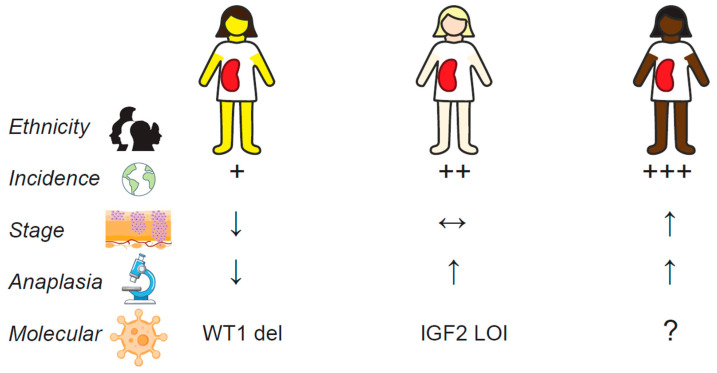
Conceptual summary of the key differences in the clinical, pathological, and molecular features of Wilms tumors in Asian, Caucasian, and African children.

In summary, Wilms tumor occurs at a lower incidence and at a younger age in Asians compared to Caucasian and African populations. Asians also appear to present at an earlier stage of disease and have a higher incidence of FH compared to Caucasians, while African children tend to present with more advanced disease. PLNR also seems to be rarely encountered in Asian Wilms tumors, which may be associated with the observed earlier age of onset in Asians. While there has been limited research into the differences in clinical and histopathological characteristics of Wilms tumors in different ethnic groups, various emerging hypotheses on inter-ethnic variations of genetic aberrations may account for the observed differences. Current studies suggest a higher incidence of WT1 deletion among Asian children compared to Caucasians and a lower frequency of IGF2 LOI among Asians. These could contribute to the lower incidence of PLNRs and tumors with unfavorable histology in Asians, but such conclusions remain supported by a limited body of evidence (Figure 2). Data from other populations outside Asia are even more lacking and further limit the inferences that can be made for these patient groups. More robust registry-based data of Wilms tumor patients from non-Caucasian populations and the characterization of their clinicopathological and molecular profiles are required to better understand biological associations with inter-ethnic differences in disease patterns. To make meaningful epidemiological comparisons, in the future, a greater inclusion of non-Caucasian patients on equivalent treatment protocols as their Caucasian counterparts will be needed.
